# Applied and implied semantics in crystallographic publishing

**DOI:** 10.1186/1758-2946-4-19

**Published:** 2012-08-30

**Authors:** Brian McMahon

**Affiliations:** 1 International Union of Crystallography, 5 Abbey Square, Chester CH1 2HU, UK

**Keywords:** Information exchange standard, Semantics, Ontology, Scholarly publishing, CIF, Crystallography

## Abstract

**Background:**

Crystallography is a data-rich, software-intensive scientific discipline with a community that has undertaken direct responsibility for publishing its own scientific journals. That community has worked actively to develop information exchange standards allowing readers of structure reports to access directly, and interact with, the scientific content of the articles.

**Results:**

Structure reports submitted to some journals of the International Union of Crystallography (IUCr) can be automatically validated and published through an efficient and cost-effective workflow. Readers can view and interact with the structures in three-dimensional visualization applications, and can access the experimental data should they wish to perform their own independent structure solution and refinement. The journals also layer on top of this facility a number of automated annotations and interpretations to add further scientific value.

**Conclusions:**

The benefits of semantically rich information exchange standards have revolutionised the scholarly publishing process for crystallography, and establish a model relevant to many other physical science disciplines.

## Background

Crystallography is a discipline with a strong tradition of good data management and cutting-edge software development, and with a community that actively values free and efficient information exchange. That community’s approach to academic publishing is presented as an example of good practice of relevance to many other physical science disciplines.

The body that governs the discipline, the International Union of Crystallography (IUCr), has published its own journals for over 60 years. It has tried wherever possible to bring active scientific content into the traditional business of publishing research literature. In the electronic publishing age, it shares the growing interest in “semantification” of the scientific literature – that is, the application of computing technologies to facilitate linking, annotation, visualization and manipulation of the text of a research publication and its associated supporting data, models and algorithms.

An early approach within the publishing industry to adding such value (*circa* 1980s) was the manual insertion, during technical editing, of additional “tags”. These were content not originally supplied by the author, but designed to be read, interpreted and executed by software responsible for typesetting the article and converting it to formats that could be displayed and manipulated on computer screens or within computer memory.

The formal system known as Standard Generalized Markup Language (SGML) [1] was developed for this purpose during the 1980s. It specified procedures for defining such tags and allowed great flexibility and extensibility of function. It also imposed an orderly structure on the document (in principle, it allowed *any* desired structure to be formally asserted and validated at run time).

SGML was not fully implemented in early electronic publishing technologies, for two reasons as relevant today as they were a generation ago. One is that computational tools to handle general metalanguage systems such as SGML are very complex to design and build. Their efficient implementation can place a heavy burden on hardware resources and on software library design. The other reason is that the real cost of adding value to publications through manual markup is very high – whether through the skills of trained editorial staff or the voluntary additional investment of time by a committed author. In particular, relying on an untrained author requires the development of authoring and editing software that is easy to use, but retains the structural integrity of the markup scheme adopted.

The first, technical, challenge has been somewhat ameliorated by the subsequent evolution of SGML. As is widely known, the ideas behind SGML were incorporated into the HTML language in Tim Berners-Lee’s initial implementation of the World-Wide Web [2,3]. They also strongly influenced the development of XML (Extensible Markup Language), a more restricted metalanguage system adopted for the development of semantic applications within many evolving Web technologies. It is also widely used in publishing. However, while this has increased the number of parsers and libraries available for software developers, it remains a challenge to design software that has general applicability across all the areas in which XML is involved. And, of course, if specialised software is written for a specific XML application, it will probably not be interoperable with programs used in other applications.

Likewise, the quest for easy authoring and editing tools continues. While the latest-generation office productivity applications (word processors, spreadsheets etc.) have user-friendly interfaces and internally use an XML model, the resultant XML is often either poorly documented or ill-suited for interoperability with other applications. These tools also suffer from the difficulty that their very ease of use hides the underlying structure of the document from the author; and in consequence the author often does not appreciate the benefits of structured information markup.

The ecosystem of scholarly publishing is in any case in a state of flux. Economic and some philosophical changes, for example the growth of open-access publication and the general availability to authors of high-quality document preparation software, are tending to place a heavier burden on authors to prepare and add semantic value to their publications. Given that many authors find this a laborious process – and that many are not expert in the necessary markup formalisms and procedures – there are parallel initiatives by publishers to enhance the semantic content of their publications through semi-automated post-processing of the submitted article.

The current article describes some of the experiences of one learned-society publisher in adding value to research publications through semantic markup and processing applied both explicitly, through a structured markup scheme, and implicitly through dynamic post-processing of specialised content in a known context.

## A formal semantic markup framework for crystallography

### Historical development

Crystallography is characterised in the *Online Dictionary of Crystallography* ( http://reference.iucr.org/dictionary/Main\_Page) as “the branch of science devoted to the study of molecular and crystalline structure and properties, with far-reaching applications in mineralogy, chemistry, physics, mathematics, biology and materials science.” It is thus an interdisciplinary science, with many sub-communities that have their own terminologies, formalisms and philosophies. However, it is fortunate in having many experimental tools and procedures in common across many of these disciplines.

In particular, the accurate determination of the three-dimensional structure of atoms and molecules in a crystal lattice, by analysis of the directions and intensities of diffracted rays from a collimated X-ray beam incident on the crystal, is a common technique widely used in fields as diverse as materials characterisation, chemical structural analysis, structural biology and structural condensed-matter physics.

The interpretation of such diffraction patterns and the deduction of the three-dimensional structures giving rise to them involve much computation, and crystallographers were early adopters of electronic computers. The relatively standard configuration of crystallographic experiments, even with different experimental probes such as X-rays, electrons and neutrons, means that it is useful to pass data from one program to another. Various *ad hoc* standard descriptions and formats of data representation sprang up as the field evolved. In due course, several integrated software suites emerged, each with its own internal data representation allowing communication between its different modular routines.

Crystallographic software developers soon became aware of the need to design data formats that could be used by many applications. They also had to address a broad collection of data descriptors (*i.e.* not solely the few variables involved in, say, an inverse Fourier transform; but also the many different types of measured data that the software could import, manipulate in various ways, and transform into derived model coordinates).

In the early days, this exercise was repeated across each distinct team of software developers. As the number of packages grew, individual crystallographers wanted to use several of them together, and to export data from one package to another.

Pressure grew for standardization, and in the 1980s the IUCr commissioned the development of a Standard Crystallographic File Structure, SCFS [4], designed not only to facilitate transfer of data between different program packages, but also to present the eventual output from the packages (three-dimensional atomic coordinates, atomic displacement parameters, descriptions of molecular geometry etc.) in a format suitable for automated incorporation into published research output.

The SCFS was modelled on the then-traditional style of formatting data in Fortran programs. Its effectiveness was limited because its release coincided with the arrival of minicomputers such as the VAX780 in crystallographic laboratories, and consequent changes in computing, in which new data types and file formats proliferated. Also, new equipment such as automatic diffractometers became standard in laboratories, each with new and different data output types and formats.

By the end of the 1980s, the IUCr considered that it was essential to develop a new standard data exchange mechanism that would break free from the constraints of fixed format, would be easily extensible to cope with new developments, and would attempt to cover all the different experimental data types that occurred across crystallography. The solution proposed by a Working Party on Crystallographic Information was named the Crystallographic Information File (CIF) [5]; a detailed history of its development can be found in the IUCr’s reference work *International Tables for Crystallography*[6].

### Adding semantics to a standard format

The earliest drafts of the new CIF standard described a simple free-form ASCII-only tag–value structure. Data items with a single value were presented as a tag (always beginning with an underscore character) separated by white space from the associated value. All “values” appeared in the file as character strings, but there were lexical rules suggesting when a particular string should be interpreted as a numerical value.

**Figure 1:**
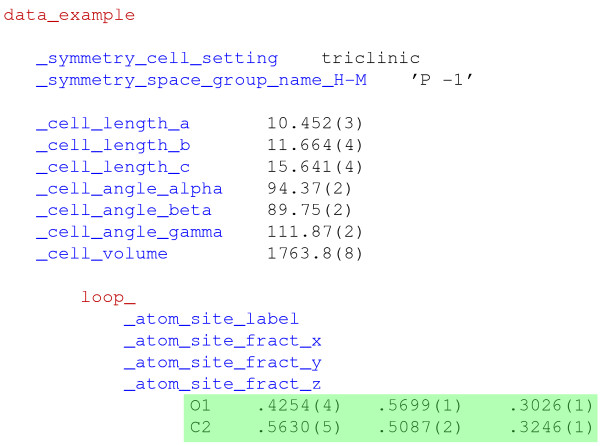
**Crystallographic data in CIF format.** This very abbreviated example illustrate the organization of data in a CIF. “Tags” (in blue) identifying the nature of discrete data items begin with an underscore character; “data” are always ASCII character strings, but will be interpreted by computer parsers as textual or numeric – trailing integers in parentheses indicate experimental uncertainty values. White space is used as a delimiter (so text strings including white-space characters must be quoted). Keywords (in red) partition major structural components of the content; the iterated values (on a green background) are tabulated in accordance with the tags listed in the loop header. Positioning of content is not relevant – the orderly layout shown here is typical of many program outputs, but is not required.

For data items with multiple values (for example vectors or matrix elements), the tag was declared once (preceded by a keyword indicating that succeeding values would be looped), and the repeated elements followed, separated by white space. Several related data items could be declared by a composite loop header, and the respective data values would be listed in interleaved sequences following the ordering of their associated tags. Figure 1 shows a small example.

To encourage rapid adoption of the new format, the initial tag set was designed to be easily human-readable, and to indicate the nature of the associated information by building each tag as an organised concatenation of keywords. Data types were to be inferred solely from lexical cues – “if it looks like a number, treat it as a number”.

However, by the time the standard was published, it was recognised that the approach was extensible to other fields, and that its wider take-up would require explicit rules for data typing, organization and constraints. For any specific application, these could be collected – in machine-readable form – in a “dictionary”. This was itself a file constructed using the same syntax rules, but it contained the information necessary for automated processing of tag sets developed for specific applications. (The file also contained explicit human-readable definitions of the concepts that each data name was associated with – part of the reason the term “dictionary” was adopted.) The particular set of tags used within the dictionaries themselves, to identify the names and usage of other tags, was known as a “Dictionary Definition Language” (DDL) [7].

In principle, one could now begin to construct rather general computer applications that had no inbuilt handling mechanism for crystallographic data, say; but which could read a dictionary of CIF tags written in DDL to learn which tags were valid, and how they might be manipulated in a crystallographic application. In similar vein, dictionaries of chemistry tags could be created using the same DDL formalism. And, in a neat closure, the DDL tags themselves could be defined in a self-defining dictionary in DDL format.

Such bootstrapping assembly of complex data structures from primitives expressed in the same formalism is not uncommon among computer metalanguages. For instance, document type definitions (DTDs) in XML applications can themselves be written in the XML language. However, the adoption of such a procedure for scientific data exchange was novel at the time CIF was proposed. Interestingly, this development was prompted by the pragmatic need to integrate crystallographic and chemical representations for organizations and applications that need to manipulate both sorts of data (such as the Cambridge Crystallographic Data Centre, which played a large role in the early development of this standard).

Such machine-readable dictionaries allowed what had been *implicit* semantic information to be replaced by an explicit set of well-defined rules that could be executed by general computer routines. Previously, a programmer had to read and interpret imprecise textual definitions of data types, and try to understand exactly how and when a tag such as _chemical_formula_weight should be used. Now these details could be recorded unambiguously and in a format that could be used directly by computer programs.

In practice, it transpired that writing software based only on the computer-readable rules was very difficult. This is true of all abstract metalanguage processors, and as suggested in the introduction, was one reason why SGML failed to become widespread. Even today many common crystallographic programs will have been written with significant manual interpretation of the CIF standard.

### New dictionaries for new purposes – involving different communities

It also transpired that the relationships between CIF data items described by these DDL terms were not detailed or rigorous enough to accommodate everyone’s requirements.

Specifically, the data model used in the first CIF dictionary provided a good description of X-ray diffraction experiments and derived structural models for small molecules or for inorganic compounds. That was a natural consequence of the route by which they had been developed, building on the structure refinement packages in common use by the small-molecule crystallographic community.

But that data model was not detailed enough to describe the complex relationships between atoms in a large biological macromolecule such as a protein. Protein crystallographers are interested in amino-acid sequences and complex folds in the structure, and how specific regions of the molecule might be related to biological activity. This type of information was already stored for structures deposited at the Protein Data Bank, and a new standard format would need at least to retain that type of information, and, preferably, to improve the efficiency and richness of the information that could be stored.

In consequence, a new, extended dictionary definition language (DDL2) was devised, over the course of a series of workshops, that could encode these data relationships (and the relationships between structure, form and function that they represented) [8]. This new formalism was adopted for the crystallography of biological macromolecules.

This has had the effect of creating two “dialects” of CIF. Although DDL2 is a superset of the original DDL (now renamed DDL1), software authors in the small-molecule community did not wish to re-factor their existing code, since the greater complexity of DDL2 brought no additional benefits. However, both dialects of CIF share a common syntax, so that many tools can handle both without any difficulty, and a reasonable degree of interoperability still exists.

At present, the CIF format is well established within the crystallographic community. For small-unit-cell structures, the DDL1-based file format has become the generally accepted standard, helped by its adoption as the sole and mandatory submission format for a number of journals (such as *Acta Crystallographica Sections C* and *E*). Many other journals (such as those of the American Chemical Society and Royal Society of Chemistry) require authors to deposit crystallographic structural models in CIF format, either with the Cambridge Structural Database or as a supplementary document in the journal archive.

Additional dictionaries have also been created to extend CIF into related fields, such as powder diffraction, electron density studies, and the description of incommensurate crystal structures. A separate dictionary provides a formalism for specifying restraints and constraints applied during refinement of the crystal structure.

The IUCr journals have played a particularly important role in the process of creating some of these dictionaries. Journal *Notes for Authors* formalise the specific information that is required or recommended for inclusion in various categories of paper, and these are very helpful in collecting the concepts that the community requires for sharing information within that area of research. For emerging subject areas, the journals have in turn been guided by recommendations from the relevant IUCr Commission. For example, the *Checklist* created by the Commission for Aperiodic Crystals [9] was an essential element in the creation of the CIF dictionary for incommensurately modulated crystal structures [10]. More recently, publication guidelines for structural modelling of small-angle scattering data [11] are likely to provide an invaluable starting point for formalising a small-angle scattering ontology.

While these new dictionaries spring up within separate communities, the IUCr provides a coordinating committee known as COMCIFS to help to harmonise their approach. COMCIFS also maintains a register of namespace prefixes that can be used by individual developers or groups to create local CIF dictionaries.

The use of such namespaces is very helpful in practice, because the art of writing precise definitions can be very difficult. There have been numerous examples of working groups established to draft CIF dictionaries, who abandon or mothball the project. Often this is because emerging scientific areas are in a state of flux over the precise definitions of still-evolving concepts and new ideas. What can then happen is that new software packages are developed that use these concepts in practical ways, and a renewed effort to draft a dictionary then has a better chance of success. CIF dictionary definitions can be initially reserved (through a namespace prefix) to a specific software implementation for purposes of test and development, which allows for competing approaches in a developing subject area. However, where interoperability between applications is important, the community will come in time to settle on a common set of terms that can be implemented across all the relevant packages.

Within structural biology, the DDL2-based mmCIF format for biological macromolecules continues to compete with other formats, since many domain applications do not need to make use of all of the information in its rich data model, and so can work with simpler formats. However, mmCIF forms the conceptual basis for the database schemas implemented within the Protein Data Bank, and is a model for ontologies expressed in other formalisms. It has also been enhanced by other structural biology communities, with extensions for structural genomics, nuclear magnetic resonance spectroscopy, cryo-electron microscopy and protein production.

Within crystallography, extensions of the DDL2-based format have also been developed for diffraction images and for a complete description of crystallographic symmetry.

In some application areas, the uptake of CIF is relatively slow. For example, detectors have historically come with their own proprietary image formats and built-in software for handling these, so that, at a purely local level, there is little need for external standards. Yet the use of the image CIF standard (imgCIF) for diffraction images allows for greater interoperability, for example within large facilities hosting equipment from different manufacturers. It also makes it feasible to consider a long-term archiving strategy for raw experimental data, and to extend a coherent workflow for data management across the whole data lifecycle, from experiment to publication. As we shall demonstrate later in this article, the greater the potential for interoperability, the more use *will* be made of the information.

### Specifying more semantic content: prospects for the future

Work is actively under way to develop an extended formalism that will allow future dictionaries to convey not only, as now, the formal relationships between distinct data items within the hierarchical organization of the data model, but also algorithmic procedures relating their mutual derivation and dependence. This would, in principle, allow the values of some missing data items to be *deduced* from the values of related data elsewhere in the file.

This formalism will be based on the DDLm (dictionary definition language with methods) approach of Spadaccini & Hall [12]. As will readily be appreciated, it provides the potential for remarkable richness in semantic markup. A general-purpose software application, written with no reference to a specific field such as crystallography, could return the value of a unit-cell volume (_cell_volume) from a file which does not contain such a value, but which has instead the values of the unit-cell edge lengths (_cell_length_a, _cell_length_b, _cell_length_c) and internal angles (_cell_angle_alpha, _cell_angle_beta, _cell_angle_gamma). Such a program will have no inbuilt procedures for calculating solid geometry; all the procedures that it needs to apply in deriving the cell volume will be specified in the dictionary definitions for the cell parameters.

If this may seem a trivial example, there are prototype implementations that are capable of calculating the complex structure factor for any reflection vector by summing over all atoms in a unit cell based on a 10-line methods definition in a CIF dictionary [13]; again, we emphasise that none of these crystallographic concepts is built into the processing engine.

The development of software that can perform extended domain-specific calculations solely from machine-specified semantic relationships is likely to be a long-term project, but implementations such as dREL [13] demonstrate that it is feasible. How well such a project succeeds will probably depend on the perceived need for general semantic querying across several disciplines. Within any individual discipline, much effort will already have gone into writing code that optimizes performance. This may involve mathematical manipulations different from those used in the reference algorithm (*i.e.* one written in a dictionary specifically to demonstrate the relationships between different quantities).

Nevertheless, the development of such dictionaries could serve a number of useful purposes. They could eliminate ambiguities in calculating values that arise from different implementations (or different understandings) of mathematical and physical formulae in different programs. For example, the multiplicity in a unit cell of atoms that are situated on “special positions” (sites through which a space-group symmetry operation passes) is calculated differently by some current crystallographic programs. They could provide benchmarking and performance functions. And they could provide independent validation of structures modelled by different software implementations. As we describe in the next section, such *validation* is already an important consequence of the semantic markup in existing CIF-based documents.

## Applied semantic markup: visualization and validation

In this section we consider two particular aspects of the improved handling of submitted structure reports arising from the semantically-rich CIF format: the ability to visualize the three-dimensional structural model (of particular use to readers of the published article) and the ability to perform independent calculations of derived information and compare with the author’s assertions (permitting in-depth technical peer review, and helping to assure the quality of the published results).

### Routine structure visualization

**Figure 2:**
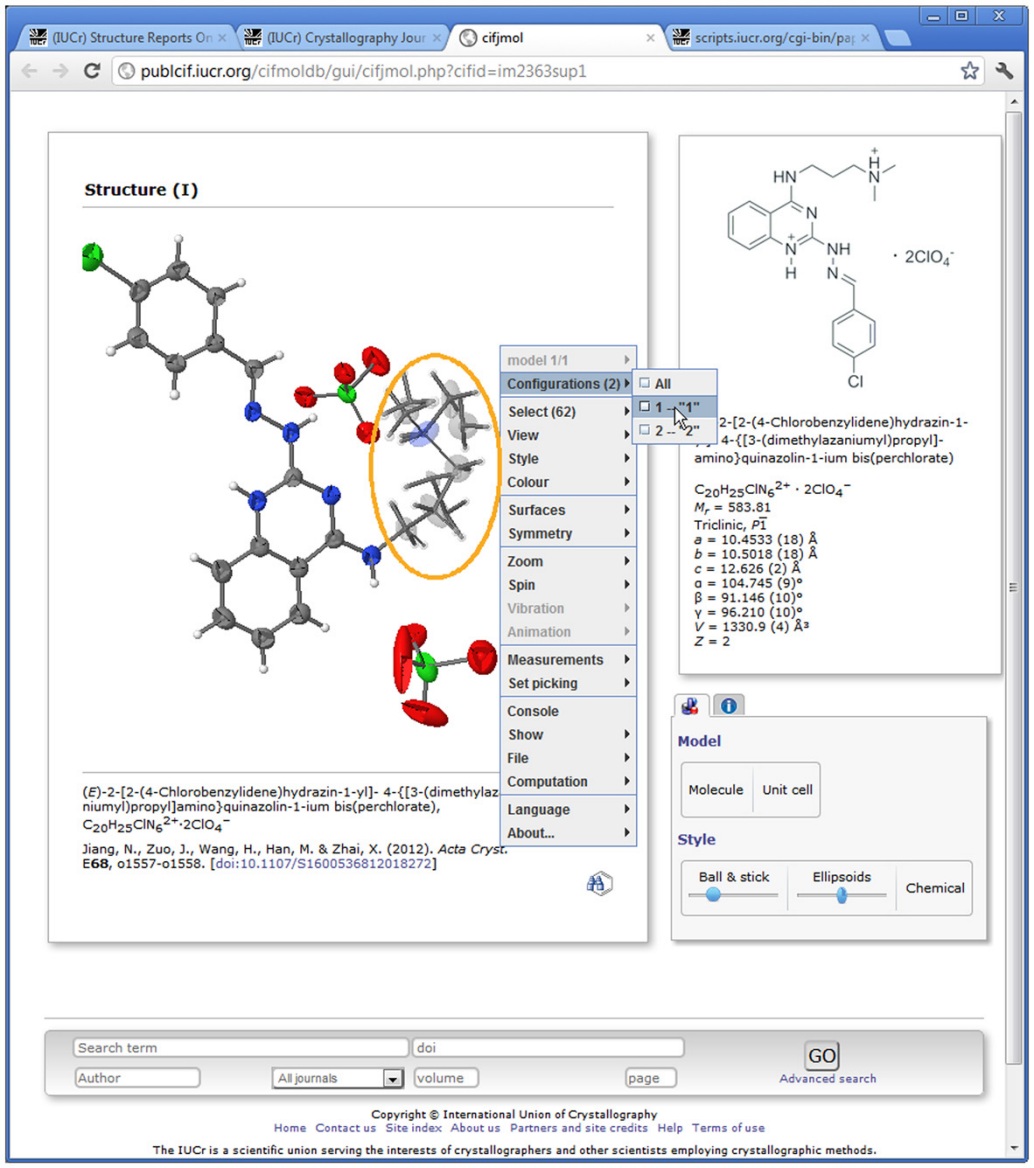
**Three-dimensional visualization of a molecular structure published in *Acta Crystallographica Section E.*** The translucent ellipsoids to the left of the pop-up *Jmol* menu (within the orange ellipse) are alternative locations of disordered atoms. The reader is about to select one of the alternative conformations and suppress display of the other (see text for full details).

For every published crystal structure in IUCr journals, we provide a three-dimensional visualization, in the form of a *Jmol*[14] applet that dynamically loads the associated CIF data set and allows the reader to interact with the model (Figure 2). Since we regard this as providing direct access to the supporting data, the “3-D view” is freely accessible from the online contents page, whether or not the actual article is available only to subscribers.

In Figure 2[15] we illustrate how the semantic markup in the submitted CIF is able to identify several of the atoms as disordered across two distinct locations. These are represented by the translucent ellipsoids on the right. This representation comes from *Jmol*, based on the information that these atoms are disordered – we emphasize that this is not some colouring or rendering scheme chosen by the author. The pop-up menu shown in Figure 2 communicates directly with *Jmol*, and provides options to display only one of the alternative disordered configurations. At the instant this screenshot was captured, the visualization is still showing the default representation (*i.e.* all the disordered sites are shown in a translucent style).

It may be noted that the pop-up menu suggests many of the other things that a reader can do using the *Jmol* tool: show the molecule in ball-and-stick or space-filling representations, change colouring schemes, show the crystal packing, measure the distances and angles between any atoms in the molecule (or crystal); as well as rotate, translate or zoom the view.

In showing the default representation, the application has already used other semantic information in the CIF to display the structure as a connected molecular model. If, instead, the structure were of an inorganic crystal, the default view would be an extended lattice with coordination polyhedra shown. If the molecule were a biological macromolecule, *Jmol* could render it as a ribbon or cartoon representation. It should be noted, however, that our journals do *not* routinely archive structural data for biological macromolecules, because of the long-standing community practice of depositing such coordinates at the Protein Data Bank (where they can also be visualized in the same manner).

### Enhanced structure visualization

**Figure 3:**
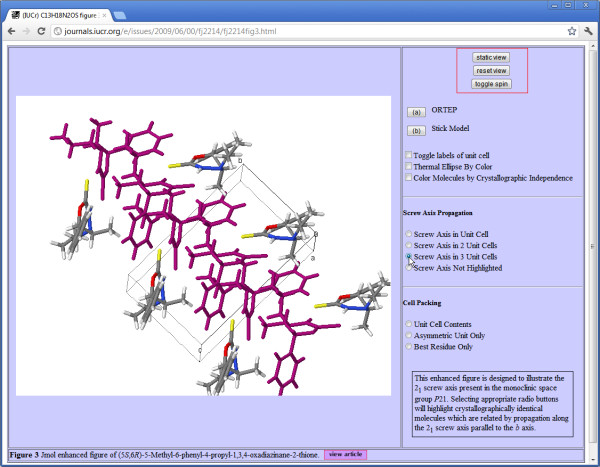
**Enhanced figure in *Acta Crystallographica Section E.*** The author has created a number of preferred views or representations of the molecule, but the reader is free also to interact directly with the three-dimensional visualization.

Since 2008, IUCr journals have also allowed authors to publish such interactive visualizations as an integral part of their research articles, and have provided a toolkit to allow authors unfamiliar with *Jmol* to use many of its powerful features [16]. Figure 3[17] is a good example of how authors can use this as a didactic tool. The buttons and check boxes on the right of this interactive figure have been constructed by the author, using the enhanced-figure toolkit, which constructs the page using HTML and Javascript snippets that the author does not see directly. These “form elements” provide distinct preferred views or representations of the structure selected by the author. In Figure 3, the reader has selected one of the available options (highlighting in maroon a group of molecules lying along a screw axis), but is also able to rotate the figure to understand better how this three-dimensional relationship appears in practice.

While this is an effective way of adding scientific value to the article, it does raise the difficulty that any particular visualization software is likely to have a limited life span. In the short term we address this pragmatically by providing an accompanying static figure, which can be swapped in if the reader’s browser platform cannot run the interactive visualization program. Such a figure is a backup device, so that at least some element of the visual information is retained for the longer term.

However, we also retain *Jmol*’s own description of the three-dimensional model it has generated from the input CIF. This is in the form of a state script, a set of commands describing the model in terms of solid objects and visual effects. Because the program is designed to describe chemical and crystal structures, the objects are described not as geometric primitives (*e.g.* “sphere”, “cylinder”), but as objects with an associated semantic function (“atom”, “bond”, “ribbon”). This, at the very least, should make it easier to implement a completely different visualization tool to render these objects in the same style if *Jmol* itself becomes defunct. It also provides a good basis for developing a standardised graphics modelling language to describe components of molecular or crystal structures in a program-independent way.

Building such a software-independent visualization language would not be an easy project, but there have been some prototyping efforts [18]. Such semantification of graphics languages would in itself be a very useful long-term goal.

### Validation of the submitted model

A traditional and laborious task of the reviewer of a crystal structure report was the independent validation of the reasonableness of the submitted model. For decades, co-editors of the IUCr journals publishing structural reports undertook such validation by hand; but as the volume of scientific research grew rapidly, this became a heavy, and ultimately unsustainable burden. With the introduction of the CIF standard, automated procedures could be introduced by the journals.

By the mid 1990s, the editorial office had a system for assembling a validation report of a submitted structure based on the output from several crystallographic applications that were by then able to read CIF directly [19]. A decade later, the analysis had been fully automated and was being provided both as an integral element of the submission and review procedures for IUCr journals, and also as a web service, *checkcif * ( http://checkcif.iucr.org), freely available for checking any crystal structure [20].

Although the analysis of a structure by *checkcif * is automated, the interpretation of the analysis still requires human judgement, and so *checkcif * has become a tool, and certainly not a replacement, for peer review. It is sometimes used by journals from other publishers; and within the IUCr, how it is used varies between journals. Yet, even with the caveat that it cannot be allowed to replace peer review, its routine use does help to make the review process more efficient.

Consider the practice in *Acta Crystallographica Section E: Structure Reports Online*. Submission of an article begins with the upload of a CIF, which is immediately subjected to *checkcif * analysis. If the analysis shows that there are “class A” or “class B” alerts (representing, respectively, extreme or significant outliers of some calculated value from the range found in most chemical structures of the same type), then the CIF *will not be admitted into the review process* unless and until it contains machine-readable comments (added by the author) referring to these outliers. These comments may simply confirm that the reasons for such outliers are discussed in the article itself; or they may provide a justification to the reviewer. In any case, they must be present, and they will be considered by the reviewer as part of the overall assessment of the quality and suitability for publication of the article. *Acta Cryst. E* also has a policy of publishing the initial validation report *and the authors’ responses to such alerts* alongside the published article. This both maximises the transparency of the review process and provides an easily digestible summary of the technical consistency of the model.

**Figure 4:**
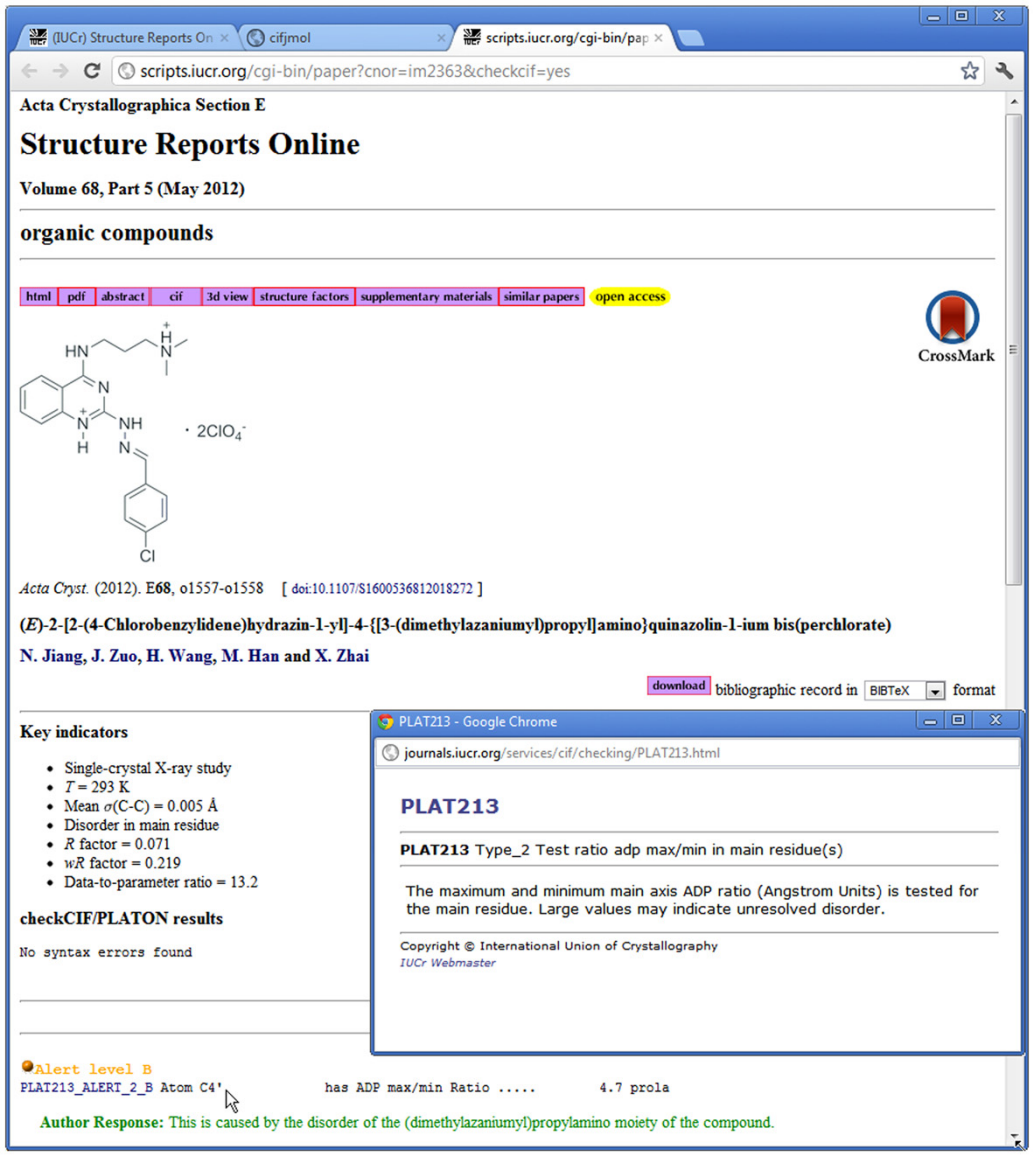
***Checkcif *validation report for the structure illustrated in Figure**2**.** The small pop-up window (towards the bottom right) has been launched as a hyperlink from the notification visible in the lower left corner of the figure.

Consider Figure 4, which is the published validation report for the article that we have already cited in Figure 2[15]. An alert at level B has been generated (near the bottom of the Figure). It has an identifying code (PLAT213_ALERT_2_B) hyperlinked to a generic explanation of the problem (in the small pop-up window), and a terse account of the issue. In this case, the atom labelled C4^*′*^ has an unusually elongated displacement ellipsoid (a.d.p.: a parametrisation of the displacement of the atom position from its mean value over all equivalent locations in the crystal lattice). The pop-up helpfully offers the comment that this may indicate unresolved disorder in the crystal. In this example, the author has responded that the unusual a.d.p. axial ratio does indeed result from localised disorder. The referee will have seen this response, and decided that it is an adequate explanation of the outlier in this case. If we refer to Figure 2 (ideally, to the actual instance of this page on the Web), we can see the problematic atom – it is the translucent grey ellipsoid just to the left of the word “Surfaces” in the pop-up menu. We see that it is within the part of the molecule where there is widespread occupational disorder, manifested in the alternative configurations that have been modelled. The ellipticity at that site is apparent in this Figure, but unless we are experts, we are unlikely to have identified it as anything out of the ordinary, without the additional help of the *checkcif * report.

What may strike the casual observer, however, is the much more pronounced elongation of the red ellipsoids (representing oxygen atoms in the solvent ${\text{ClO}}_{4}^{-}$ (perchlorate) ion at the lower right). The *checkcif * procedure has not considered this ion, since it is common practice for such solvent ions not to be fully refined. This illustrates the extent to which the analysis can take full account of context, given the complete crystallographic information in the submitted CIF.

## Implicit semantics in a structure report article

As mentioned in the Background section, it is expensive (in terms of effort and expertise) to add fully semantic markup to documents. For crystal structure reports, there is a great deal of semantic markup in the CIF – happily, most of it “written” by the computer programs used in the reduction and analysis of the data. But in the free-text part of the article, embedded in the CIF as distinct data fields, we do not expect authors to mark up their commentary in any greater detail than in a conventional article. Nevertheless, from the *context* of this commentary, we are able to make more reliable assumptions and inferences about the subject matter that allow the journals to overlay additional semantic functionality. In this section we explore a few examples of how this is done.

### The virtual hyperglossary

In our simplest example we describe how we annotate the textual content of any of our journal articles, not just structure reports.

It is common for online web pages to highlight terms and phrases and hyperlink them to definitions or expanded explications in online dictionaries. Over-zealous annotation can irritate the reader, if every occurrence of a common noun is highlighted; but judicious annotation can help non-expert readers better to understand the content of technical documents. An early proposal to create servers that would annotate documents in this way was put forward by Murray-Rust and West during the 1990s [21] under the name of *virtual hyperglossary*.

We provide such a virtual hyperglossary for all our online articles. As each article is served, the reader may select whether to activate such annotations. If such an annotation is required, the web server scans each page, using a variable-length window that progresses through the document (initially four words, then three, two and one) to compare phrases against a master index. Where a match is found, the phrase is hyperlinked to the target associated with that indexed phrase – usually a definition from the *Online Dictionary of Crystallography* ( http://reference.iucr.org) or IUPAC *Gold Book* ( http://goldbook.iupac.org). The index uses “stop words” to exclude common words, or to annotate them in certain cases (see the following discussion relating to the word “group”).

The search/match procedure is simple and fast; and it is effective, because we are able to constrain the target terminologies to those most appropriate to the material we publish – *i.e.* to crystallography or chemistry definitions. This is simply because we *only* publish in those domains; and we can construct relevant mappings because we can tune the look-up tables to reflect common patterns of usage.

For example, we do not routinely hyperlink the single word “group”, which can have many meanings. We do identify phrases such as “space group” and “plane group”, which are specific to crystallography, and link them to their definitions in the *Online Dictionary of Crystallography*. However, we can also scan for phrases such as “associative group” or “group theory”, or even phrases that do not contain the word “group”, such as “inverse element”, and link these directly to the entry for “group” in the *Online Dictionary* (which is a definition specifically of a mathematical group).

This is, of course, a rather simple example. We select an annotation resource based solely on the general subject matter covered by our journals. Nevertheless, it is a context-specific choice that can add real semantic value and a significant level of extra utility for the reader.

### Embedded geometry references

Our second example is a little more interesting, because it mines the active semantic markup in the current document to provide a context for subsequent inferences.

**Figure 5:**
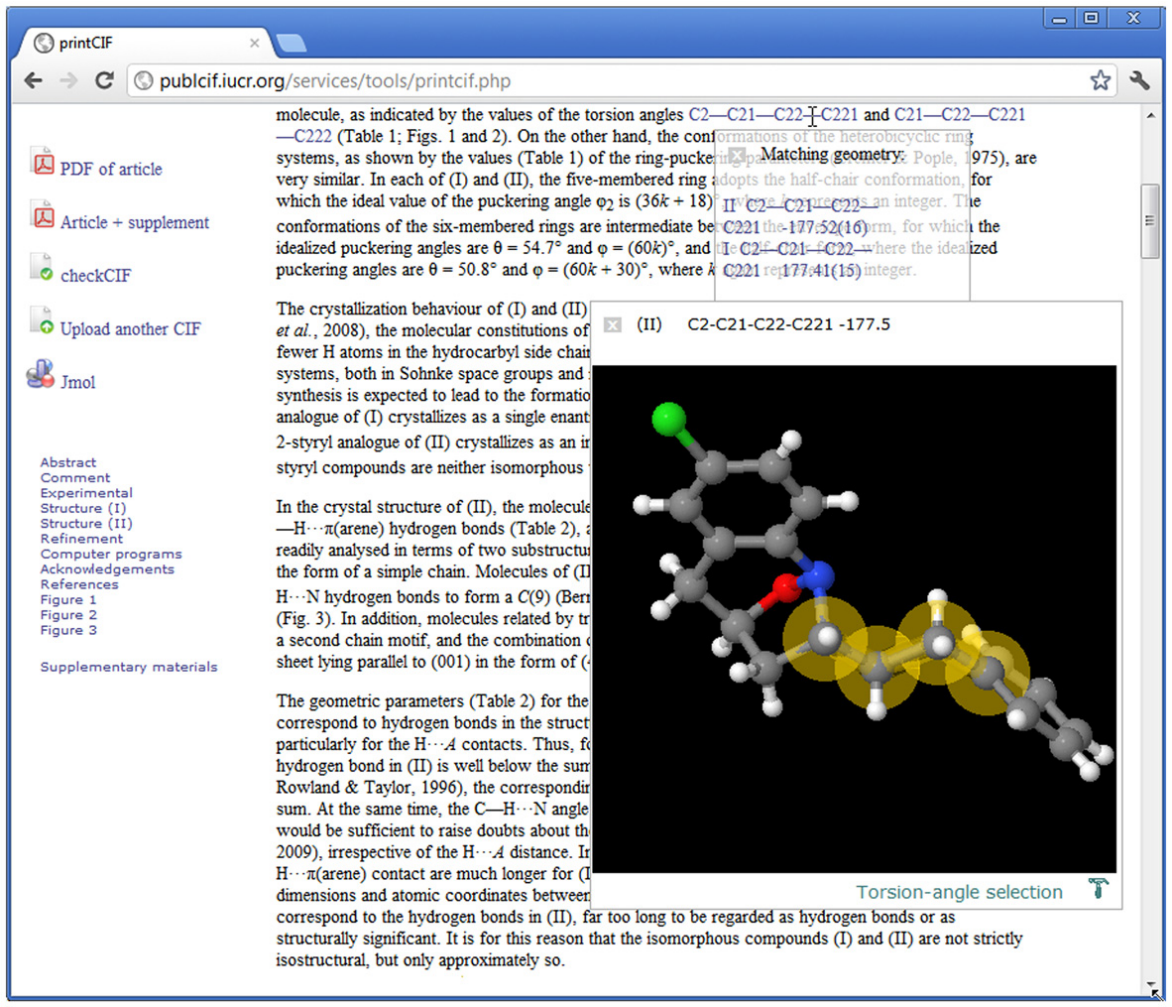
**Visualization of an angle referred to in the discursive text.** The pop-up *Jmol* window highlights the torsion angle selected on the top line of the page area (see text for full details).

Figure 5 shows a rendering of a page from one of our journals [22] in a tool used by authors to preview their article before submission. In the top line of the text, some torsion angles are referenced. The reader has placed the mouse over the first of these, and a pop-up window identifies two torsion angles with these atom labels (they occur in the two separate structures discussed in the article). The reader selects the one found in compound II, and a *Jmol* window appears with the selected angle highlighted. Again, within this window the reader is free to modify the view, say by generating the crystal packing to explore exactly where that torsion angle is located in the crystal lattice.

The text in the article has *not* been additionally marked up. However, a simple scanner detects regular expressions characteristic of molecular geometry (bond distances, angles, torsions or hydrogen-bond contacts). Using these typographic cues, a lookup can be performed of the actual geometric values that *are* listed with full semantic tagging elsewhere in the CIF. If a single match is found, the visualization will immediately show that; if there are multiple matches, as in this example, the reader is asked to select the feature of particular interest.

This approach is more discriminating than a heuristic based *solely* on typographic cues – candidate geometric properties must not only have the correct typographic form (*e.g.*C2---C21---C22---C221) but must also correspond to a tabulated angle or bond involving the labelled atoms (C2, C21 etc.). It is an approach that offers significant additional semantic information for a large proportion of the published literature, without additional markup effort from authors or editors.

### Chemical structure deduced from crystallography

As our final example, we look at something more challenging – handling the sometimes complex relationship between a crystallographic and a chemical description of a molecule with limited semantic information. Consider again Figure 2. Alongside the three-dimensional representation of the molecule, derived from the crystallographic experiment, there is a two-dimensional chemical diagram that displays molecular connectivity, bond types, assigned charges, and sometimes stereochemistry.

Current practice is to obtain this chemical diagram from the author as a graphic (*e.g.* TIFF file). While this provides rich visual information, the chemistry cannot reliably be extracted for computational purposes from an image file. A better approach would be to require a machine-readable description of the chemistry, for example by requiring that the author provide a .mol or .sdf file.

We do not make this a requirement, in part because there may be resistance from the author (this is not a widespread practice throughout chemistry generally); and in part because it would add to the validation requirements for each submission. Because the available file formats are proprietary, have limitations on the features they can reliably show, and use different atom labelling schemes from crystallographic programs, it is difficult to devise automated validation procedures, such as those in place for the crystallography.

**Figure 6:**
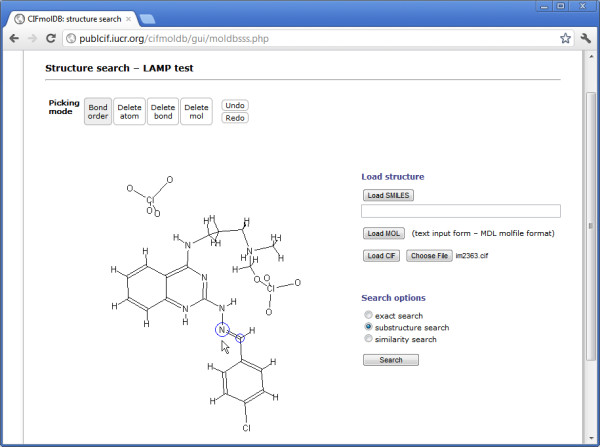
**Building a chemical representation of a molecule from its crystallographic description. ***Jmol* is here being used as a chemical editor. The wireframe model is actually a projection onto the plane of a stylized three-dimensional structural view.

We are therefore investigating the possibility of providing a tool for authors to construct a fully machine-readable description of the molecular chemistry that is derived initially from the crystallographic model (Figure 6). In this example, the same CIF that was used in Figure 2[15] forms the basis for a chemical description of the molecule. Compare with the chemical diagram in Figure 2. The two-dimensional layout is not as tidy as the author’s supplied diagram, because here it is a projected view of a three-dimensional structure. The bond assignments shown are those determined by the *Jmol* loading script (based on standard chemical values), but the author now has the opportunity to correct any mis-assignments.

As the structure is corrected, so the application can generate a connectivity graph that may be used for substructure and similarity searching of chemical databases (interfaces for such searches may also be seen in Figure 6). At the same time, the connection table may be written back into the CIF, using a formalism that has existed from the initial release of the core CIF dictionary.

However, while this procedure works very well for small or simply-connected organic molecules, there are greater challenges in handling large metal–organic complexes, or polymers. This is, of course, not a surprise – standard chemical databases have a variety of approaches to storing descriptions of such types of compound, and the description that is stored in a particular case may depend substantially on the interpretation of the editor loading that structure into the database.

On a number of occasions the CIF community has explored how (indeed, even whether it is possible) to determine a unique and unambiguous chemical description of a molecular assembly from its three-dimensional packing in a crystal lattice. It is not an easy project, and so far there has been no success in extending the chemical structural description in the core CIF dictionary to accommodate the most complicated cases. It is a particularly difficult problem where there is significant crystallographic disorder.

The conclusion, therefore, is that chemical semantics describing a molecule *can* be inferred from a crystallographic description in many cases; but that for complex molecules, implied semantics do not suffice – the explicit interpretation of an expert must be added to the otherwise well-defined crystallographic description.

## Ontologies and pragmatics

It is not uncommon these days for informatics-driven applications to begin with a formal ontology, frequently specified in a standard representation such as OWL [23]. As mentioned in Section “Background”, CIF pre-dates the development of such ontologies, but its pragmatic adoption of dictionaries and the increasing semantic richness of those machine-readable dictionaries provide an opportunity to compare its ‘bottom-up’ formalism with the ‘top-down’ approach of starting with an ontology.

The early CIF dictionaries supplied elements of taxonomy and controlled vocabulary for the interchange of specific data between computer packages. Since these packages all shared a common conceptual space (description of an X-ray diffraction experiment and its derived structural model), this was sufficient for interoperability. The new standard also imposed certain constraints on programmers; for example, all physical quantities had to be expressed in the preferred unit stated in the dictionary. Such constraints were accepted, if not always cheerfully, by programmers in the discipline, and so the range of concepts formalised in the CIF dictionaries could be kept within certain limits.

Although these decriptions were optimised for use by crystallographers, their completeness (given an *a priori* understanding of the context in which they would be used) made them sufficiently portable that they could be used as the basis for the tags describing structure in Chemical Markup Language (CML) [24].

Where the crystallographic applications overlapped to a greater extent with more general data management, it did become necessary to provide a richer description of the relationships between conceptual classes handled by the dictionary. In structural biology, the macromolecular (mmCIF) dictionary has a strong ontological flavour [25], in which, for example, scale factors and relationships between physical units are tabulated. This will allow physical quantities in mmCIF format to be automatically scaled or compared with physical quantities in other applications (although the mmCIF standard continues to mandate that only one preferred unit is actually used for each physical quantity).

More substantially, the mmCIF dictionary introduces a classification scheme (‘category groups’) that collect together the multiple data names that together comprise the description of a conceptual class. For example, the ‘entity’ group describes the class of discrete chemical entities (*e.g.* separate molecular moieties), while the ‘chem_comp’ group describes chemical components (*e.g.* amino-acid residues within a polymeric protein molecule). The importance of maintaining separate classes to differentiate such functional and conceptual distinctions has been emphasised in the construction of the ChemAxiom ontologies [26].

In strictly crystallographic applications, the CIF dictionaries present a rather ‘flat’ abstract data model. That is, the ontologically rich schema in the mmCIF dictionary is isomorphic to a relational database schema. This is not coincidental, as the protein structure entries in the Protein Data Bank are stored in a relational database management system, and this is adequate even for describing secondary structural motifs within proteins. In many applications in chemistry, this is not an appropriate or efficient data model. However, the STAR File format [27] (which supplies the syntactic structure of CIF) allows for more complex data models (closer in organisation to that of an object-relational database), and there are applications in other fields that use an extended STAR syntax within an ontological framework also described by CIF dictionaries. The Molecular Information File (MIF) [28] describes chemical (including Markush) structures, while NMR-STAR, used in the BioMagResBank database of NMR structures [29], describes ensembles of NMR models. These have associated dictionaries constructed using DDL1 and DDL2 formalisms, respectively, and are used alongside CIF data files within the Cambridge Structural Database and Protein Data Bank respectively. Because of the different underlying syntaxes, interoperability between these formats does require some additional work to achieve. Nevertheless, it is clear that CIF is largely compatible with many existing complementary data representations.

The amount of software engineering that is necessary to permit interoperability between systems based on different ontological frameworks should not be underestimated. Nevertheless, such technical modifications are well within the skill set of competent informatics scientists and software architects. It is in the actual definitions collected in CIF dictionaries, their organisation and classification by domain experts, that the efforts of the crystallographic community have resulted in the most important asset for future semantic mining and applications.

## Concluding remarks

We have demonstrated in our section *Applied semantic markup* that rich tagging of data files provides robust approaches to visualization and validation. As a consequence, the quality of structures published in IUCr journals has improved overall; but, more importantly, the *actual* quality of an individual structure (which can depend on factors outside the experimentalist’s control, such as the susceptibility of a compound to X-ray radiation damage) can be objectively assessed. The structure can be easily explored – features of geometry that the author has referenced can be checked, and other geometric details as easily evaluated and explored. Specific data can be extracted from the description and re-analysed or re-purposed in different applications.

In our section *Implicit semantics in a structure report article*, we demonstrated how the publication process can overlay additional semantic information on top of that explicitly provided within an article. At its simplest, we can make certain assumptions concerning words and phrases in the article, based solely on the fact that publication in one of our journals circumscribes the topics that are likely to be discussed.

For the case of structure reports where there is access to semantically tagged data, certain elements of the discussion in an article can be keyed to the relevant data values, allowing additional functionality (such as visualization, hyperlinking or structure searches) to be embedded in the article.

In the case of inferring chemical structure, we demonstrated that some semantic enrichment is possible; but, increasingly, the reliability of such inferences will diminish as the complexity of the data increases.

In the short term, the publisher’s ability to add such value through “implied semantification” is of benefit, and does reduce the burden on the author of preparing a manuscript for submission. However, we hope we have indicated some of the shortcomings of such an approach. In spite of the richness of structured information presented in a CIF, there remains scope for ambiguity and error, and we would wish to see progressively more semantic markup, along with the development of user-friendly tools so that authors come to regard this as a normal part of the publication process.

Key to all these developments has been the development of CIF as an information interchange standard, and its adoption throughout the crystallographic community. This has made the basic generation of semantic articles straightforward and relatively painless for authors of structure reports. By providing desktop tools such as *publcif *[30], and web services for authors such as those illustrated in the current article, the IUCr is working to further increase the explicit semantic markup in its article submissions, and consequently the usefulness and reusability of its scholarly publications. But already we have succeeded in changing the research article from an inert record of a scientific experiment to a living publication, delivering straight to the researcher the scientific results, their supporting data, and the ability to interact and critically interrogate those data. We look forward to seeing similar developments in other areas of the physical sciences.

## Competing interests

The author is employed as the Research and Development Officer of the International Union of Crystallography.
